# Development of an Electromagnetic Micromanipulator Levitation System for Metal Additive Manufacturing Applications

**DOI:** 10.3390/mi13040585

**Published:** 2022-04-09

**Authors:** Parichit Kumar, Saksham Malik, Ehsan Toyserkani, Mir Behrad Khamesee

**Affiliations:** Department of Mechanical and Mechatronics Engineering, University of Waterloo, Waterloo, ON N2L 3G1, Canada; parichit.kumar@uwaterloo.ca (P.K.); saksham.malik@uwaterloo.ca (S.M.); ehsan.toyserkani@uwaterloo.ca (E.T.)

**Keywords:** magnetic levitation, additive manufacturing, eddy current levitation, direct energy deposition

## Abstract

Magnetism and magnetic levitation has found significant interest within the field of micromanipulation of objects. Additive manufacturing (AM), which is the computer-controlled process for creating 3D objects through the deposition of materials, has also been relevant within the academic environment. Despite the research conducted individually within the two fields, there has been minimal overlapping research. The non-contact nature of magnetic micromanipulator levitation systems makes it a prime candidate within AM environments. The feasibility of integrating magnetic micromanipulator levitation system, which includes two concentric coils embedded within a high permeability material and carrying currents in opposite directions, for additive manufacturing applications is presented in this article. The working principle, the optimization and relevant design decisions pertaining to the micromanipulator levitation system are discussed. The optimized dimensions of the system allow for 920 turns in the inner coil and 800 turns in the outer coil resulting in a $N_{inner\;coil}$:$N_{outer\;coil}$ ratio of 1.15. Use of principles of free levitation, which is production of levitation and restoration forces with the coils, to levitate non-magnetic conductive materials with compatibility and applications within the AM environment are discussed. The Magnetomotive Force (MMF) ratio of the coils are adjusted by incorporation of an resistor in parallel to the outer coil to facilitate sufficient levitation forces in the axial axis while producing satisfactory restoration forces in the lateral axes resulting in the levitation of an aluminum disc with a levitation height of 4.5 mm. An additional payload of up to 15.2 g (59% of mass of levitated disc) was added to a levitated aluminum disk of 26 g showing the system capability coping with payload variations, which is crucial in AM process to gradually deploy masses. The final envisioned system is expected to have positional stability within the tolerance range of a few μm. The system performance is verified through the use of simulations (ANSYS Maxwell) and experimental analyses. A novel method of using the ratio of conductivity (σ) of the material to density (ρ) of the material to determine the compatibility of the levitation ability of non-magnetic materials with magnetic levitation application is also formulated. The key advantage of this method is that it does not rely on experimental analyses to determine the levitation ability of materials.

## 1. Introduction

In recent times, magnetism has garnered significant interest within academic spheres. Magnetism has found applications in various fields, such as the damping capability [1], levitation and manipulation of a magnetized object [2], energy harvesting [3], sensing applications [4] and manipulation of magnetorheological fluid (MRF) [5] among several others, owing to its compatibility and adaptability to these applications.

Magnetic levitation is a promising field that brings forth an alternative contact-free method of moving objects. The absence of the physical aspect of actuation opens avenues for its applicability within both the macro and micro manipulation applications. Within the macro-scale, magnetic levitation has found applications in transportation [6] and wireless charging [7] among several others.

There has been significant research associated with the development of magnetic systems within the micromanipulation sphere. Ref. [8] discussed the development and actuation of bio-inspired robots, which mimic the motion style of different micro-organisms and are actuated using the principles of magnetism.

Ref. [9] studied the use of a magnetic micromanipulator levitation system to provide a wireless mode of navigation for surgical devices and drug containers within the human body using the principles of magnetism. Ref. [10] studied the development of micro-robots working on the principle of magnetic levitation. Ref. [11] studied the characteristics of a floating magnet freely levitated between two diamagnetic places without an external energy input.

Ref. [12] highlighted the use of magnetic fields for the actuation and micromanipulation of dynamically self-assembled magnetic droplets. Ref. [13] discussed the design, fabrication, actuation and control of versatile micro and nano robots with significant applications within the biomedical industry, while also discussing the bio-safety aspects of the system. Despite the extensive research in the field, a manipulator capable of supporting levitation with stability in the micrometer (μm) range and compatible with additive manufacturing environment has not been developed.

Additive manufacturing (AM) has also been a primary point of emphasis for research within the academic environment. The prevalence of AM in fields, including tooling, repair and reconditioning [14]; the aerospace industry [15]; medical implants [16] with a special focus on the development of metallic implants [17]; dental devices [18]; and the micro-fabrication of microrobots [19], has significantly strengthened the importance of the field. However, despite the vastness of and the emphasis placed on the two fields, there is minimal to no overlap between magnetism and AM.

Metal AM processes are reliant on the delivery of feedstock materials, such as sheets, powder or wire on a substrate/object, melting the feedstock materials using a reliable energy source, such as lasers, electron beam/arc among others, and the subsequent solidification of the material in a layer-by-layer approach [20]. The ability to make complex parts using metals makes it a prime candidate for further research.

The two most commonly used techniques for metal additive manufacturing are laser powder fed additive manufacturing (LPF-AM) (also known as the direct energy deposition (DED) technique) and the laser powder bed additive manufacturing (LPB-AM) technique (also known as powder bed fusion (PBF) technique). The primary emphasis is placed on LPF-AM technique due to its compatibility with the electromagnetic micromanipulator levitation system discussed in this research. The critical components of interest within the LPF-AM system are typically a high energy laser system, a powder feeding component/nozzle and a movable worktable [21] or robotic arm [22].

The LPF-AM technique generates tool paths for deposition using sliced cross-sectional dimensions obtained from CAD models. The material nozzle head carries the material (usually powder form) along with an inner gas (e.g., argon) to deliver the feedstock material onto the build surface. Through the use of a power source (e.g., laser), the material deposited is melted and solidified to build parts using the layer-by-layer approach as described in [23].

According to [24], the critical process parameters associated with the implementation of LPF-AM technique arise from two critical steps: The delivery of heat and mass onto the build surface and the interaction of the heat and mass at the build surface. The parameters of relevance include the laser source ignition, gas-powder delivery, powder stream and laser beam interaction and melt pool formation by heat conduction, among several others. These parameters aid in determining the quality of parts built with LPF-AM. As discussed in Section 6, the key process parameters to determine the compatibility of the electromagnetic micromanipulator levitation system are the mass deposition rate, the velocity of powder and the nozzle angle.

The design for the electromagnetic micromanipulator levitation system discussed in this paper was developed with the objective of creating a compact arrangement of electric coils to be used in an AM environment. The specific application of this system correlates with a LPF-AM type of metal 3D printing where a computer-controlled robotic arm melts a metal filament with a laser or an electron beam and create complex structures.

The use of magnetic micromanipulator levitation system for AM applications offers some significant advantages. First, building the part on a levitated geometry bypasses the need for a substrate as utilized in conventional AM applications [25]. Furthermore, since the levitated geometry is anticipated to be a portion of the built part, the amount of postmanufacturing operations required is reduced significantly. A proof of concept for the application of magnetism and acoustic levitation systems for AM application was developed [26]. However, the article presents a high-level view of the idea.

Designing an Electromagnetic (EM) micromanipulator levitation system is not quite straightforward as multiple factors need to be considered. The material selection, geometric constraints, input power and peak frequency capabilities, undesirable inductance and impedance related drawbacks are only a few of the major criteria that need to be evaluated. This article offers a comprehensive analysis of such a system, comparing simulation analyses conducted in ANSYS Maxwell, a world-renowned EM simulation software, with the experimental apparatus developed. Key emphasis is placed upon the constraints that led to the development of the system causing the above-mentioned drawbacks and the strategies employed to overcome them.

Material properties have been a crucial point of emphasis for magnetic levitation applications. Conventional magnetic levitation techniques focus on the material properties of the core [27] or the ferromagnetism of the levitated part [28]. The novel magnetic levitation technique presented in this article aims to use the principle of eddy current levitation to facilitate the levitation of nonmagnetic materials, such as aluminum.

While this technique has been developed previously [29,30], the emphasis has always been placed on the levitation of aluminum. However, the compatibility of other conducting materials for eddy current applications has not been explored. Ref. [31] presented the compatibility of various conductive metals for induction heating applications. They developed the levitation ability of materials as the ratio of the maximum levitation force ($F_{lev}$) to the weight of the object (*W*) as presented in Equation (1).

(1) $$Lev\;Ability=\frac{F_{lev}}{W}$$

However, Ref. [31] relied heavily on the use of experimental analyses to measure the maximum levitation force to develop the levitation ability of the materials. The research presented in that article aimed to develop a parameter to ease the process of determining the levitation ability of materials and subsequently the compatibility of different conducting materials for eddy current levitation applications. The theory presented was verified through simulation analyses.

**Figure 1:**
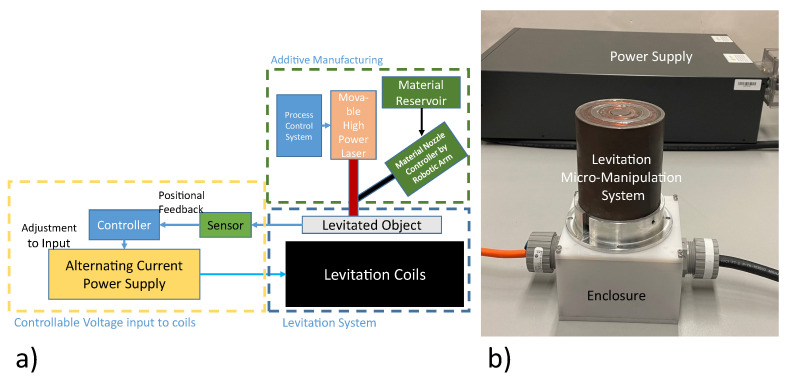
Micromanipulator levitation system. (**a**) Schematic for the envisioned system. (**b**) Experimental apparatus.

The objective of the research presented in this article is the development of a magnetic micromanipulator levitation system that is suitable for AM applications. The schematic for the envisioned system is shown in Figure 1a. The initial constraints, optimization and associated design decisions, implementation through simulation and experimental analyses and the compatibility of the developed system with AM applications are presented. The final envisioned manipulator is expected to provide a positional stability of a few micrometers in the axial and lateral axes. This positional stability is anticipated to be offered with the effects of powder deposition as well.

## 2. Theory

### 2.1. Working Principle

The working principle of the micromanipulator levitation system was described in our previous work [32] as follows.

- According to Ampere’s Law, the time varying currents of the coil will produce time-varying magnetic fields
- According to Faraday’s Law, the axial component of the time-varying magnetic fields will induce a voltage in conductors placed in close proximity to the micromanipulator levitation system. This will result in the induction of currents in the conductors. These induced currents are called eddy currents.
- According to Lorentz’ Law, the induced eddy currents will interact with the radial component of the source magnetic field to generate repulsive force. This resulting force will be responsible for the levitation of the levitated object.

Additional details of the working principle are presented in Section 2.5.

### 2.2. Electromagnetic Micromanipulator Levitation System

The micromanipulator levitation system is the set of electromagnetic coils responsible for the generation of levitation force resulting in the free suspension of the levitated disc as shown in Figure 1b.

The two concentric coils carry current in the opposite directions. The inner coil is responsible for the production of levitation force in the axial (*z*) axis while the outer coil is responsible for the production of restoration forces in the lateral (*x*,*y*) axes to facilitate levitation at the equilibrium point. The two coils are placed within a highly magnetized iron core to facilitate magnetic focusing towards the levitated geometry.

### 2.3. Micromanipulator Levitation System as a Series RLC Circuit

Impedance is defined as the measure of overall opposition of the circuit elements to the current flowing through it. Impedance is different from conventional resistance because impedance is variable with frequency. Higher frequencies result in high impedance. Impedance is also significantly dependent on the inductance and capacitance of the circuit as well. The relationship of these components is shown in Equation (2). Inductive reactance (shown by $X_{L}$) is the opposition to the current offered by the inductor in the circuit. Capacitive reactance (given by $X_{C}$) is the opposition to the current offered by the capacitor in the circuit.

(2) $$Z=\sqrt{R^{2}+{\left(X_{L}-X_{C}\right)}^{2}}$$

$$X_{L}=2\pi fL\;,\;X_{C}=\frac{1}{2\pi fC}$$

where *R* is the *DC* resistance of the circuit, $X_{L}$ is the inductive reactance, $X_{C}$ is the capacitive reactance, *f* is the frequency of supplied voltage, *L* is the inductance of the circuit, and *C* is the capacitance of the circuit.

Impedance becomes quite relevant in applications with alternating currents. Thus, a key point of emphasis of this research was to minimize the overall impedance of the system. The lower the impedance of the system, the higher the current across the coils with a given voltage input. Thus, minimizing the impedance results in an increase in the current across the levitator, which subsequently results in higher levitation forces.

### 2.4. Magnetomotive Force of Coils

The force of levitation is directly affected by the Magnetomotive Force (MMF) of the coils. The MMF is the product of the number of turns of the coils to the current through the coils [33]. According to [29], the average force of levitation is derived as:

(3) $$F_{Z_{avg}}=\frac{{\left(N1-N2\right)}^{2}I_{0}^{2}\mu_{0}A_{airgap}}{4z^{2}}$$

where *N*1 and *N*2 are the number of turns of turns of coil 1 and coil 2, respectively, $I_{0}$ is the current through the coils, $\mu_{0}$ is the permeability of free space, $A_{airgap}$ is the area of the air gap under the disc, and *z* is the distance of the disc from the levitation coil.

N×I is the magnetomotive force, which is the line integral of the magnetic intensity around a closed line. Maximizing N×I is the objective function of the development of this system, while minimizing the inductance ‘L’, as well as the resulting impedance ‘Z’, which is the cost function. A higher resultant levitation force is a necessity to counteract the opposing force (weight) imposed by the deposition of material on the disc substrate in an AM environment. The inductance of a multi-coil, multi-core system is a non-linear property, and theoretical calculation of the same is not an objective for this research, it will be calculated and minimized through simulations using ANSYS Maxwell (Version: ANSYS electronics desktop 2020 R2. Pennsylvania, USA.

### 2.5. Levitation Ability

The working principle described in Section 2.1 are modelled analytically as shown in Equations (4) (Faraday’s Law), (5) (Ohms Law) and (6) (Lorentz’ Law):

(4) $$E_{induced}=\frac{d\phi }{dt}$$

(5) $$J=E_{induced}\;\frac{\sigma A_{conductor}}{l}$$

(6) $$F_{lev}=\int J\times BdV$$

where *J* is the induced eddy currents, ϕ is the magnetic flux, *B* is the magnetic field, $F_{Lev}$ is the levitation force, $E_{induced}$ is the induced emf, *l* is the length of conductor, $A_{conductor}$ is the area of the conductor, σ is the conductivity of the material and *dV* is the differential volume of the conductor. The gravitational force of the levitated object is modelled as in Equation (7).

(7) $$F_{gravity}=mg=\rho Vg$$

where *m* is the mass of the object, ρ is the density of the material of the conductor, *V* is the volume of the levitated object, and *g* is the gravitational constant. According to [31], the levitation ability of a material is the defined as the ratio of the maximum levitation force experienced by the levitated object to the gravitational force experienced by the levitated object. This is described analytically in Equation (5).

According to [31], the levitation ability of a material is the defined as the ratio of the maximum levitation force experienced by the levitated object to the gravitational force experienced by the levitated object. This is described analytically in Equation (8).

(8) $$Lev\;Ability=\frac{F_{lev}}{F_{gravity}}=\frac{\int J\times BdV}{\rho Vg}$$

As can be seen from Equations (4)–(6), the only material property that affects the levitation force of the conductor is the conductivity of the material. As evident from Equation (7), the only material property of the material that affects the gravitational force on the object is the density of the material. Thus, the levitation ability of materials can be represented as the ratio of the conductivity to the density of the material as shown in Equation (9).

(9) $$Lev\;Ability=\frac{F_{lev}}{F_{gravity}}=\frac{\int J\times BdV}{\rho Vg}\;\alpha \;\frac{\sigma }{\rho }$$

**Figure 2:**
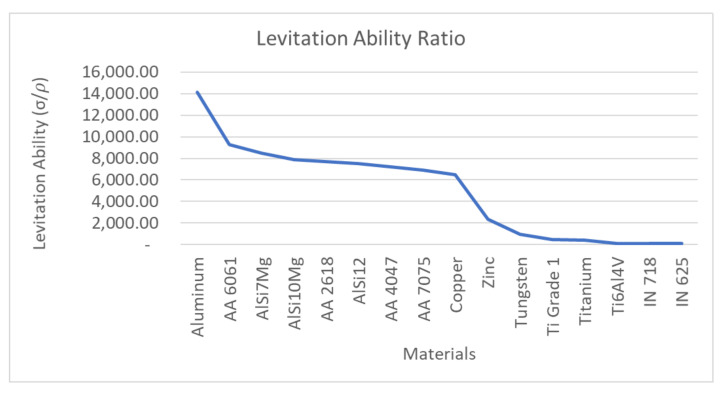
Levitation ability: the ratio of $\frac{\sigma }{\rho }$ for different materials.

Several different materials were considered for the analysis. A strong emphasis was placed on materials used for additive manufacturing applications. The technique is critical to determine the compatibility of different materials employed for AM operations with the magnetic micromanipulator levitation system. The levitation ability of several conductors is plotted in Figure 2. According to Figure 2, aluminum and its alloys have a high levitation ability owing to the high conductivity and low density. Other materials, such as titanium and nickel and its alloys have a low levitation ability due to their high density.

## 3. Design and Optimization of the Micromanipulator Levitation System

**Figure 3:**
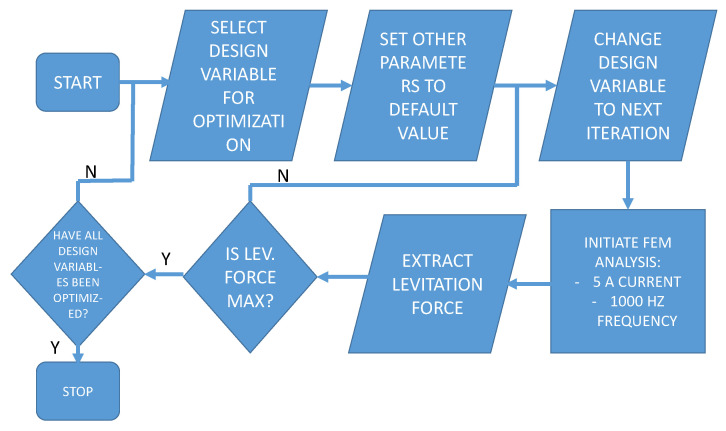
Flowchart of the optimization technique.

The optimization technique used here is the trial-and-error direct substitution method. As shown in Figure 3, each design variable is selected individually, and the design variable is iterated within a range of values, keeping every other parameter constant. The value of the design variable with maximum levitation force output is selected as the optimized value. The optimization procedure entails the use of finite element analysis (FEA) software ANSYS Maxwell to compute the objective function (which is the levitation force in the axial axis).

**Figure 4:**
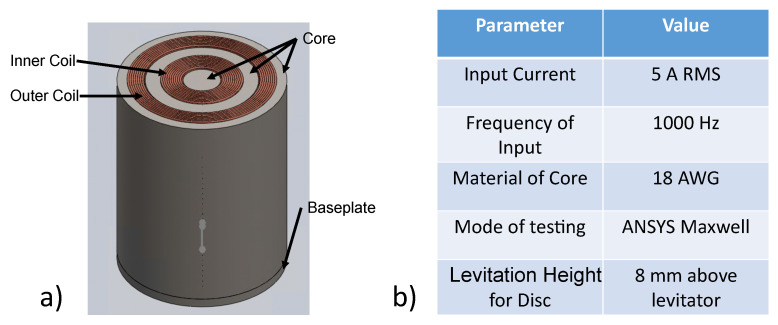
System components and parameters of micromanipulator levitation system. (**a**) CAD Model of the envisioned System. (**b**) Definition of constant parameters for optimization.

The levitator setup (with system components as shown in Figure 4a) is supposed to be placed within an AM machine. Thus, there was a dimensional constraint that the outer diameter of the system could not exceed 90 mm in the radial axis. This constraint was developed using the DMD-IC106 AM machine as reference. The DMD-IC106 has a working envelop of 350 mm × 350 mm × 350 mm [34]. For the developed system to be minimally invasive, the volume occupied by the magnetic micromanipulator levitation system was minimized. By placing a 90 mm constraint on the system, the final levitation system, consisting of the coils and its enclosure, has a total volume 11,735,850 mm${}^{3}$. Thus, the levitation system only occupies 28% of the available working envelope, therefore, leaving sufficient volume for the material deposition activities.

The levitated object in consideration is a disc of radius 25 mm and height 5 mm. Some of the parameters kept constant throughout the optimization process are shown in Figure 4b.

### 3.1. Optimization of Width of Coils

**Figure 5:**
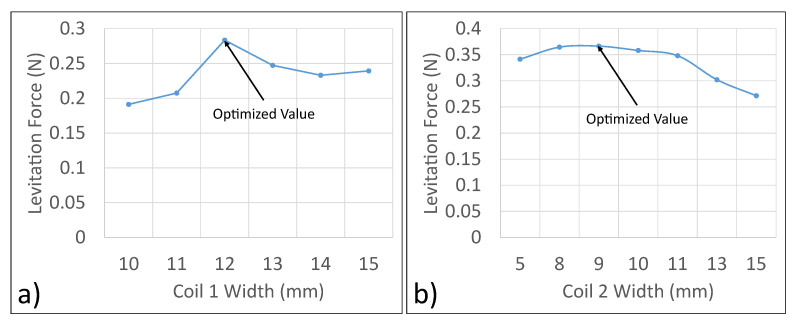
Optimization of the width of coils. (**a**) Optimization of the width of Coil 1. (**b**) Optimization of the width of Coil 2.

The trend of levitation force as a function of the coil 1 width and coil 2 width is presented in Figure 5a and Figure 5b, respectively. From the data obtained, the optimized width of the inner coil was 12 mm, and the optimized width of the outer coil was 9 mm.

### 3.2. Optimization of the Radial Placement of Teh Coil

**Figure 6:**
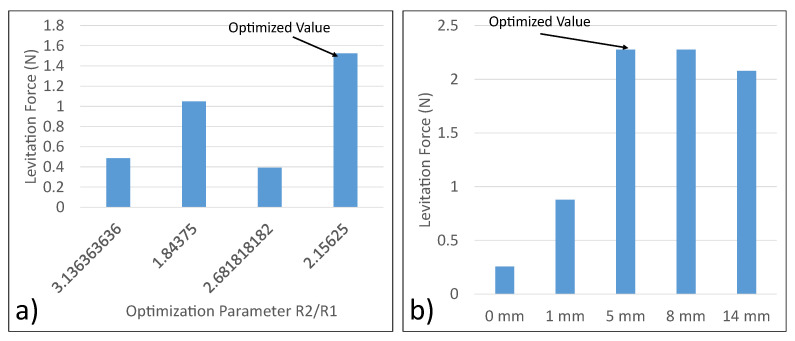
(**a**) Optimization of radial placement of coils; (**b**) Optimization of baseplate height.

Following the optimization of the two coil widths, we determined the placement of these coils within the highly magnetized ferrite cores. The ratio of the mean radius of coil 2 (R2) to the mean radius of coil 1 (R1) was used as the optimization parameter. The trend of the levitation force as a function of R2/R1 was study to determine the optimum placement of the coils. The resulting plot is shown in Figure 6a. As shown in Figure 6a, the optimum R2/R1 is 2.1562 (R2 = 34.5 mm, R1 = 16 mm).

### 3.3. Calculation of Coil Height

Having computed the width of the coils, the radial placement of the coils and the gauge of the wire to be used, the fill factor (*FF*) of the coil was used to determine the height of the coil. The fill factor is the ratio of the area of electrical conductor (cross-sectional area of wire) to area of the provided space (cross-sectional of coil) as shown in Equation (10).

(10) $$FF=\frac{d^{2}\cdot \frac{\pi }{4}\cdot n}{b\cdot h}$$

where *d* is the diameter of the wire, *n* is the number of conductors in the coil, *b* is the width and *h* is the height of the coil. For the sake of this analysis, a fill factor of 0.72 was used.

(11) $$\begin{array}{cc} 0.72 & =\frac{\frac{\pi }{4}\;{\left(1.1\right)}^{2}\;1000}{12\;h_{coil1}}\\ h_{coil1} & =110\;\mathrm{mm} \end{array}$$

To keep the same height for coil 1 and coil 2, the number of turns for coil 2 were adjusted as shown in Equation (12).

(12) $$N=\frac{110\times (0.8)\times 9}{\frac{\pi }{4}\times {\left(1.1\right)}^{2}}=750.05∼750\;textturns$$

During the coil manufacturing process, with the incorporation of epoxy and potential variations in the assumed vs. real wire diameter values, the numbers of turns that could be incorporated in coil 1 and coil 2 were 920 and 800, respectively.

### 3.4. Optimization of Baseplate

A baseplate is a plate attached to the bottom of the micromanipulator levitation system. The key objective of the baseplate is to improve the magnetic field focusing capability of the system. Five different cases were considered for the analysis here. First, there was no baseplate added to the levitator. Next, 1 mm, 5 mm, 8 mm and 14 mm baseplates were added. The levitation forces for the cases were compared. The case with the maximum levitation force was pursued as the final optimized levitator setup. As shown in Figure 6b, the levitation force is maximum for the 5 mm baseplate.

### 3.5. Selection of Core Material

The next step was to find the ideal material for the core. Ferrite, electric steel and pure iron were shortlisted due to their high permeability. Solid cores were compared. Comparisons are based on levitation force and restoration force (with 5 mm displacement in x axis). The analysis was also conducted at 60 Hz to get a deeper understanding of the material performance.

**Figure 7:**
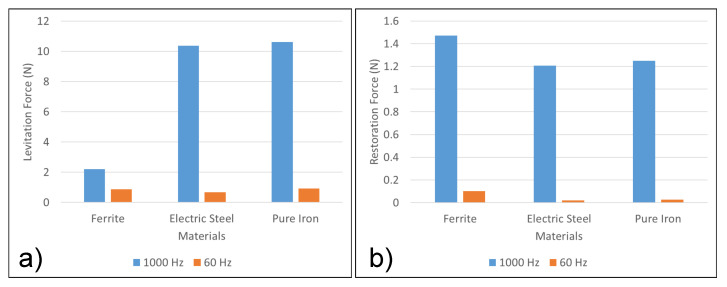
Comparison of performance of the materials. (**a**) Comparison of core materials: Levitation Force (N). (**b**) Comparison of core materials: Restoration Force (N).

As evident from Figure 7a, pure iron cores produce the highest levitation force. Figure 7b shows that pure iron also produces comparable restoration forces at both 1000 and 60 Hz. The final system parameters are listed in Table 1.

Pure iron (99.6% pure) was utilized to serve as the core of the micromanipulator levitation system. The BH curve, which is the graph plotted between magnetic flux density (B) and the magnetic field strength (H), of the material is from the specification sheet of the material [35]. For the range of magnetic field anticipated for the micromanipulator levitation system (<1000 G), the magnetic permeability of the core was found to be 700.

### 3.6. Frequency of Operation

**Figure 8:**
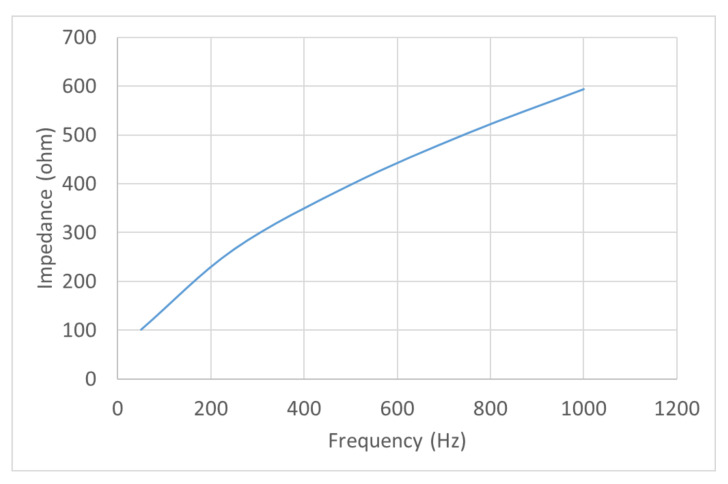
Impedance vs. frequency for the micromanipulator levitation system from ANSYS Maxwell.

Due to the dimensional constraints of the micromanipulator levitation system developed, the height of the micromanipulator levitation system needed to be high to incorporate the number of turns for coil 1 and coil 2 as explained in Section 3.3. This resulted in a significant increase in the inductance (and subsequently the impedance). This is highlighted through the variation of impedance vs. frequency for the micromanipulator levitation system obtained from ANSYS Maxwell as shown in Figure 8. To facilitate operation through a voltage controlled power supply, a frequency with a low impedance output was selected for operation.

## 4. Free Levitation Experiment

### 4.1. Initial Levitation Experiment

**Figure 9:**
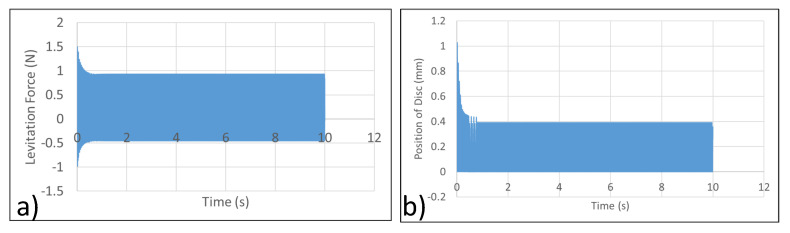
Simulation data from ANSYS Maxwell. (**a**) Levitation force vs. Time. (**b**) Position of disc vs. Time.

As discussed in Section 2.2, a set of two concentric coils carrying current in the opposite directions was employed for the levitation experiment. Some of the key characteristics of the system are shown in Table 1. ANSYS Maxwell was used for the simulation analyses. The simulation analysis of the proposed system is shown in Figure 9. According to the simulation data obtained, a current micromanipulator levitation system apparatus attempting to levitate a disc with a 25 mm radius will not be able to do so successfully. The simulation data was verified through experiments, where high-frequency vibration of the levitated disc was observed.

### 4.2. Strength of Coils

Since the two coils carry current in the opposite directions, through the principle of superposition, the two coils oppose each others magnetic fields. From theory, it is known that the inner coil is responsible for the production of axial levitation force while the outer coil is responsible for the production of restoration forces in the lateral axes as outlined in Section 2.1. Since sufficient force is not observed in the axial axis, there was a need to reduce the strength of the outer coil to facilitate a higher levitation force in the axial axis.

As explained in Section 2.4, the strength of the coils is dependent on two factors: The number of turns of the coil and the current through the coil as shown in Equation (13).

(13) $$Coil\;Strength=\frac{N1\;I}{N2\;I}=\frac{N1}{N2}$$

The current ratio of the strength of coils of 1.15 produces significant restoration forces in the lateral axes. However, the system is unable to produce sufficient levitation forces in the axial axis to facilitate free levitation. Since the number of turns were established before-hand, the strength of coils was altered by changing the current through the coil.

**Figure 10:**
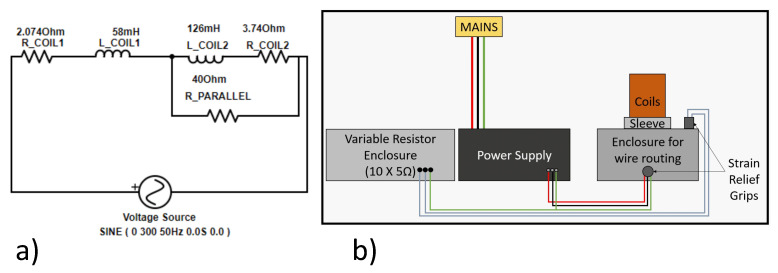
(**a**) Circuit representation of the coils + resistor in parallel setup. (**b**) Schematic of the experimental apparatus with adjusted strength of coils.

From the initial experiment, it was clear that the strength of the outer coil need to be reduced to facilitate an increase the levitation forces in the axial axis. This was achieved through the addition of a resistor in parallel to coil 2. The circuit setup for the system is shown in Figure 10. As expected, the current through coil 2 would split between the coil and the resistor in parallel. Through simulation and experimental analysis, steady state levitation was with a 40 Ω resistor in parallel to coil 2 at an operating frequency of 85 Hz.

### 4.3. Simulation Analysis

**Figure 11:**
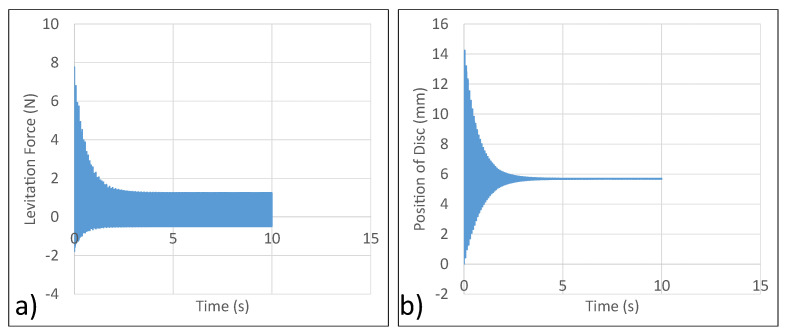
Simulation result with the resistor in parallel (**a**) Levitation force vs. time—with resistor in parallel. (**b**) Position of disc vs. time—with resistor in parallel.

ANSYS Maxwell was used for the analysis. A 300 V input at 85 Hz was supplied to the micromanipulator levitation system. With the 300 V at 85 Hz input, the current through coil 1 (I1) is 4.6582 A RMS and the current through coil 2 (I2) is 2.7844 A RMS. The levitation force in the axial axis and the position of disc as a function of time were extracted from the simulation results. The data are presented in Figure 11.

### 4.4. Experimental Analysis

**Figure 12:**
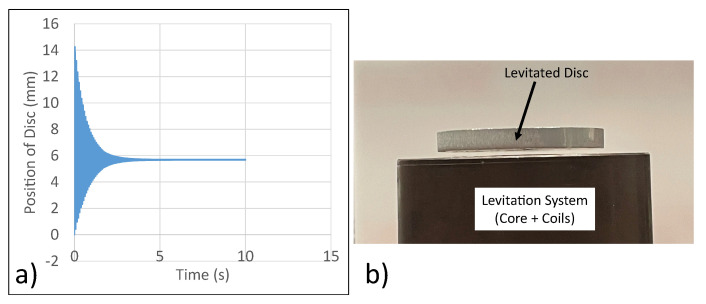
Experimental verification of analysis (**a**) Position of Disc vs. Time—ANSYS Maxwell. (**b**) Steady State Position of Disc—Experiment.

To verify the simulation analysis conducted, a 300 V RMS at 85 Hz was supplied to the levitation coils. The power supply employed for the experiment was BK precision Model 9830B, which can support an RMS power output of 3000 VA (10 A RMS at 300 V RMS) with a response time of 1.5 ms and the frequency of operation between the range of 43–1000 Hz. The steady state position of the disc was then compared to the data obtained through simulations. The experiment shows the steady state position of the disc to be about 4.5 mm. This is in close agreement with the simulation data. Thus, the experiment was deemed a success as shown in Figure 12. Thus, steady state levitation of the disc was achieved.

### 4.5. Experimental Analysis with Additional Payload

A critical aspect of AM is the addition of material on the substrate as a function of time. Thus, it was critical to depict the retention of stability of the levitated substrate with the addition of mass as a function of time. In order to highlight the systems ability to support mass added during the AM operation and its subsequent compatibility within the AM environment, an experiment was conducted to mock up AM environment where the mass of the levitated geometry will increase with time.

**Figure 13:**
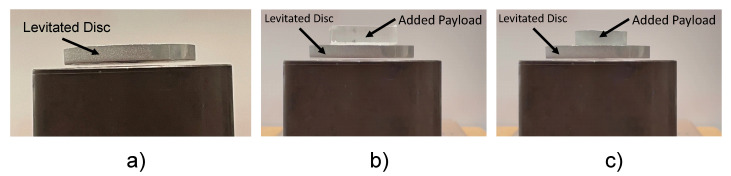
Experiment with additional payloads to highlight stable levitation with added mass as expected within the AM environment. (**a**) Free levitation of aluminum disc. (**b**) Added payload: 4.4 g (17% of initial disc). (**c**) Added payload: 15.2 g (59% of initial disc).

The following experiment was conducted to ensure the system can cope with payload variation in AM environment. Here, two additional distinct masses were added to the levitated disc and its behavior was observed. Two different payloads (15.2 and 4.4 g) were placed on top of the levitated substrate to observe the system’s ability to retain stability. Following the addition of the payload, steady state levitation was observed for both payloads as shown in Figure 13.

## 5. Semi-Levitation Experiment

### 5.1. Need for Semi-Levitation Experiment

With the successful implementation of free levitation of a suspended disc, the emphasis was shifted to a critical drawback of the system: the range of disc radii that can be supported for free levitation. With the increase in disc radii, the weight of the disc increases and will thus require a larger strength of coil for the inner coil.

**Figure 14:**
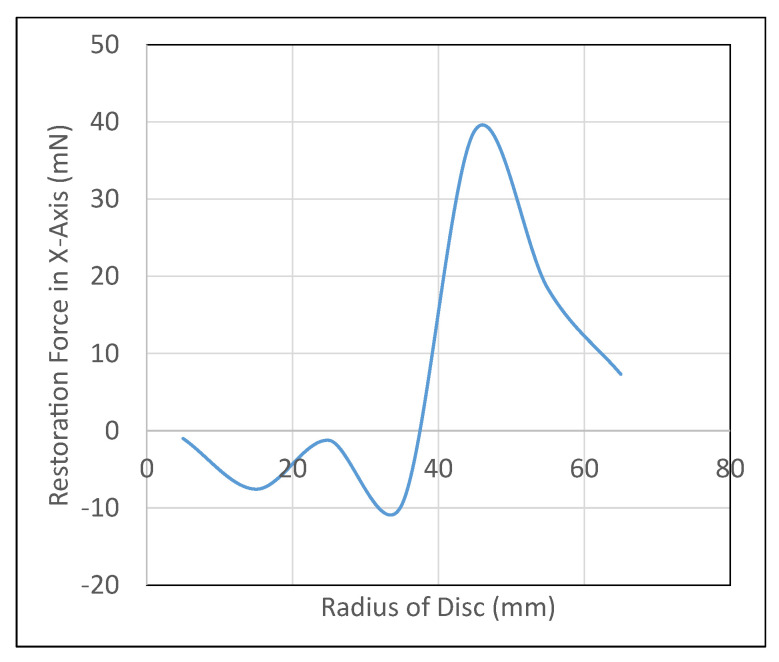
The need for a micromanipulator semi-levitation system.

In addition, the disc will lose its lateral stability as disc radius increases. This was verified through the employment of ANSYS Maxwell simulation analyses. Figure 14 shows the variation of restoration forces (obtained from ANSYS Maxwell) in the lateral (x) axis with a 3 mm displacement provided in the positive x-axis. As it can be seen, for the radii of disc over 40 mm, the forces in the x-axis are positive, therefore, contributing negatively to the stability of the system.

### 5.2. Working Principle

The two coils carry currents in the same direction to maximize the levitation forces. Since the two coils carry current in the same direction, through the principle of superposition, the effect of the magnetic field of the system will increase. However, since the outer coil also carries current in the same direction as the inner coil, no restoration forces will be produced in the lateral axes. To counteract the loss of stability, the system uses 4 external stands that serve as supports to provide restoration forces in the lateral axes.

### 5.3. Simulation and Experimental Analyses

**Figure 15:**
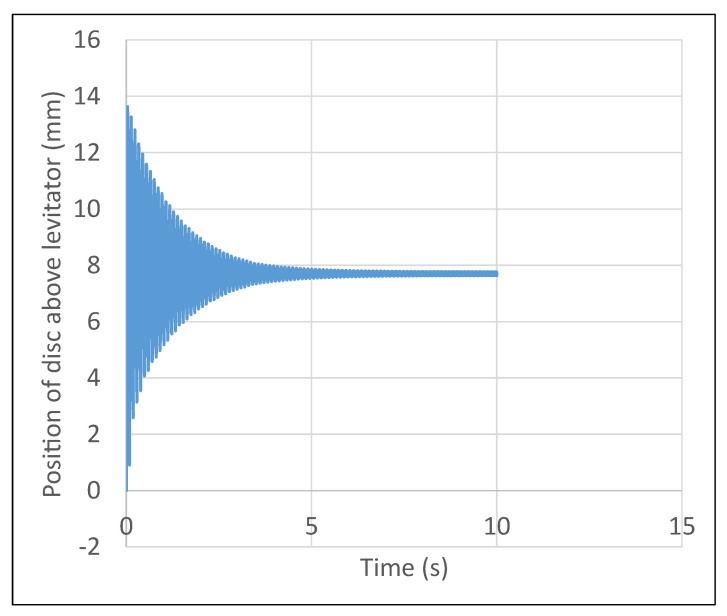
Performance of micromanipulator semi-levitation system.

A 300 V RMS input at 50 Hz is supplied to the coils. First ANSYS Maxwell is used to extract the variation of position of disc as a function of time. Next, the levitation coils are connected in series carrying current in the same direction. The resulting data from ANSYS Maxwell is shown in Figure 15. From simulation, the steady state position of the disc was 7.8 mm. From experimental analysis, the steady state position of the disc was 8 mm. Thus, simulation and experimental analysis are in close agreement.

## 6. Compatibility of Micromanipulator Levitation System with Additive Manufacturing Applications

**Figure 16:**
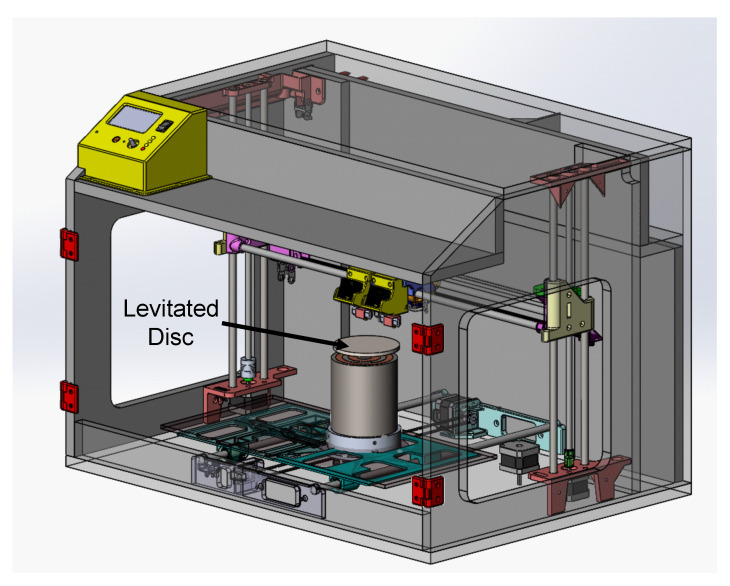
Envisioned MagLev system within the AM enclosure.

The envisioned interaction of the magnetic micromanipulator levitation system within the AM enclosure is shown in Figure 16. Having successfully conducted the free levitation and semi-levitation experiment, it was integral to determine the compatibility of the magnetic micromanipulator levitation system with AM applications. Section 4.5 presents the experimental verification of the levitated substrate capable of supporting added payload as a function of time without losing stability. The compatibility of the micromanipulator levitation system is further tested by accounting for the effect of powder deposition.

### 6.1. Context

In Laser Powder-Fed AM, the powder stream is fed onto the substrate and fused through the use of lasers on the substrate. The levitated geometry is expected to be subjected to a similar process. Thus, the deposition of powder during the AM process can have a detrimental impact of the stability of the levitated geometry. According to [25], some of the critical AM process parameters are:

- Mass deposition rate: 5 g/min [25].
- Velocity of powder: 2 m/s [36].
- Nozzle angle: 60 degree [25].

Two analyses are conducted to verify the stability of the system with powder deposition.

### 6.2. Axial Stability

The first step is to verify that sufficient levitation force is produced to overcome the effect of powder deposition in the axial axis. To verify the performance, a worse case scenario analysis is conducted to ensure reliable performance during real operation. Some of the critical assumptions for this analysis are:

- The analysis assumes steady flow of powders and the impact of air friction is negligible. Since AM operations occur in a vacuum, this assumption is fair.
- Since the size of the particles are very small and originating at the same source, the collisions between the particles can be ignored.
- The nozzle angle is 0 degrees. This ensures all the force of powder deposition only acts in the axial axis.
- The coefficient of restitution is 0. This implies that all the kinetic energy of the powder is transferred to the levitated geometry and the final velocity of the powder is 0 m/s.
- The mass deposition is assumed to be continuous at 5 g/s (higher than the expected mass deposition rate)

The principle of transfer of momentum is highlighted in Equation (14).

(14) $$F_{impact}=m_{powder}\left(V_{powder,final}-V_{powder,initial}\right)$$

Inputting the data presented in Section 6.1, the impact force results are highlighted in Equation (15).

(15) $$F_{impacts}=0.005\left(0-2\right)=0.01\;N$$

**Figure 17:**
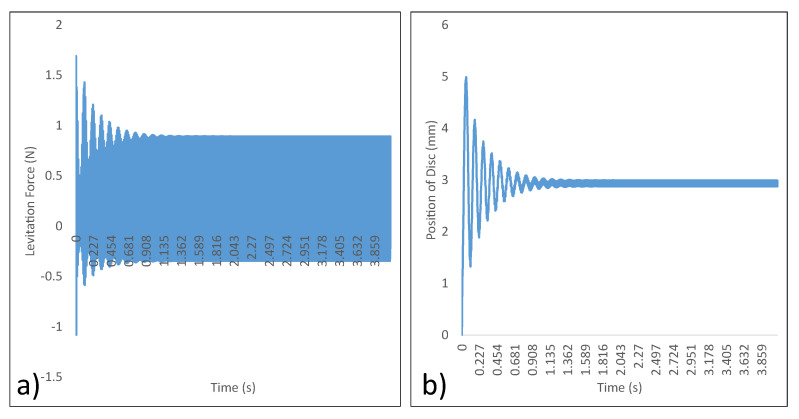
Stability of the disc in the axial axis (**a**) Levitation force vs. time (**b**) Position of disc vs. time.

Adding the force of continuous deposition along with the force of gravity in ANSYS Maxwell, it can be clearly seen that the system retains its stability. The system stabilizes 3 mm above the levitator as shown in Figure 17. It should be noted that a worse case scenario analysis was conducted with a highly exaggerated mass deposition rate. Highlighting the system’s ability to facilitate stable suspension in the worse-case-scenario analysis shows that the system will allow for stable suspension with real operation as well.

### 6.3. Lateral Stability

**Figure 18:**
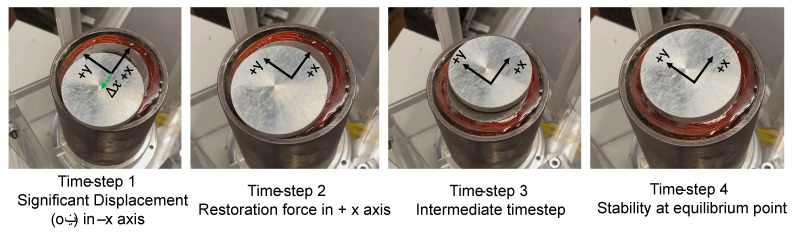
Stability of disc in the lateral axis.

Lateral stability of the system was verified experimentally. Here, the micromanipulator levitation systems ability to come back to its equilibrium point was tested by displacing the disc significantly (20 mm displacement) in the lateral axis. Upon turning the micromanipulator levitation system on, the disc moves back to its expected equilibrium position. This is highlighted in Figure 18, which shows the variation of position of disc vs. time, with the achievement of steady state levitation at the equilibrium point.

From the experiment, it can be clearly seen that the system is capable for maintaining stability even with large displacements in the lateral axes. The forces of impact of powder deposition will not cause displacements in the lateral axes larger than 20 mm. Thus, the stability of micromanipulator levitation system will be retained in the axial axis with powder deposition.

## 7. Verification of Levitation Ability

The semi-levitation experimental apparatus is utilized for the verification of the levitation ability of materials. Ref. [31] states that the maximum levitation force should be emphasized for the development and verification of levitation ability of materials. With the two coils in series, the magnetic field is maximized through the principle of superposition. Thus, the levitation force produced is maximized in the semi-levitation experimental apparatus. The levitation force on the disc is documented 2 mm above the levitation. A 300 V RMS input is supplied to the coils at a frequency of 50 Hz.

Simulation analyses are relied upon for the verification of the levitation ability of materials. Through simulations, a wide variety of materials with applications in the AM environment could be subject to testing. The use of several materials will bolster the validity of the principle of levitation ability. The reliability of simulation analyses has also been verified in Section 4 and Section 5 by depicting the close agreement between simulation and experimental analyses.

**Figure 19:**
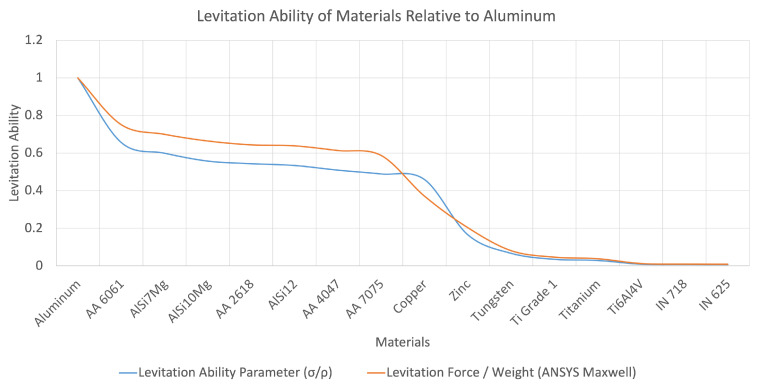
Levitation ability. Analytical model, simulation.

The analytical model and simulation data for different materials have been normalized relative to the levitation ability of aluminum. The ratio of levitation force to gravitational force (weight) was obtained through ANSYS Maxwell. The data are documented in Figure 19. As it can be seen, the two plots follow the same trend. Thus, the viability of the principle of the levitation ability of materials was verified.

## 8. Conclusions and Future Work

The simulation and experimental work shown in this article highlights the initial viability of electromagnetic levitation of materials for additive manufacturing applications. The optimization of the micromanipulator levitation system and most relevant design decisions are discussed. To facilitate levitation using the magnetic micromanipulator levitation system, a resistor was added in parallel to the outer coil to reduce the MMF of the outer coil, therefore, facilitating levitation of the geometry that was to be suspended. With a 300 V input at 85 Hz input and a 40 Ω resistor in parallel to the outer coil, an aluminum disc of 25 mm radius and 5 mm height was levitated at a height of 4.5 mm, thereby, highlighting successful levitation with the system.

The compatibility of the micromanipulator levitation system developed with AM applications is also discussed in great detail. Three distinct analyses were conducted to verify the compatibility. First, the ability of the micromanipulator levitation system to support the suspension of an additional payload (to mimic the effect of added mass due to mass deposition in the AM processes) was tested experimentally. The system was able to support the suspension of the initial geometry plus up to 15.2 g (59% of the initial geometry mass).

Next, the effect of the impact of powder deposition, which is critical to the AM process, on the stability of the levitated geometry was reported. Through simulation analysis, the levitated geometry retained its stability in the axial axis despite having a powder deposition force of 0.01 N (which is much higher than the expected force of powder deposition) acting on the same. The system also produced sufficient restoration forces in the lateral axes, which was highlighted through experiments. The levitated geometry was given a significant 20 mm initial displacement (as a disturbance) in the x-axis.

The levitated geometry subsequently came back to the equilibrium point once the system was turned on. The novel methodology of using the ratio of conductivity of the levitated geometry’s material (σ) to the density of the material (ρ) to determine the compatibility of different materials within the AM sphere with magnetic levitation applications was also discussed. The newly developed method relies solely on material properties for the determination of levitation ability of materials as opposed to experimental analyses. The testing of the micromanipulator levitation system within the AM enclosure to satisfactorily build a part using the technique LPF-AM is the critical next step of this research. The testing of the compatibility will be conducted using the DMD-IC106, an industrial metal 3D printer. Some important factors to consider include ensuring that no collision exists between the components of the AM machine and the micromanipulator levitation system, ensuring the micromanipulator levitation system is protected from the intrusive conductive dust present in the AM machine, and ensuring that no damage occurs to the laser due to laser-back reflection (LBR). LBR occurs when the light from the laser beam reflects back into the optic resulting in optical result loss and damage to the equipment.

Next, the positional stability offered by the micromanipulator levitation system will be verified with and without the effects of powder deposition. The final envisioned system is expected to provide a positional stability of a few micrometers (μm). The development a set of process parameters for deposition of the selected alloy on the levitated part to arrive at high-quality printed components is also critical. This includes parameters, such as the laser power, powder feed rate and nozzle angle, to facilitate optimum AM operations.

## Figures and Tables

**Table 1:** Specifications of the levitation system.

Parameter	Value
Outer Diameter of Levitation System	90 mm
Height of Levitation System	115 mm
Core Material	Pure Iron
No. of turns Coil 1 N1	920
No. of turns Coil 2 N2	800
Distance between disc and levitator	0 mm
Wire AWG	18 AWG
Disc Radius	25 mm

## Data Availability

Data available from the authors upon request.
